# Characterization of the Pathophysiological Role of CD47 in Uveal Melanoma

**DOI:** 10.3390/molecules24132450

**Published:** 2019-07-04

**Authors:** Maria Cristina Petralia, Emanuela Mazzon, Paolo Fagone, Andrea Russo, Antonio Longo, Teresio Avitabile, Ferdinando Nicoletti, Michele Reibaldi, Maria Sofia Basile

**Affiliations:** 1 IRCCS Centro Neurolesi Bonino Pulejo, C.da Casazza, 98124 Messina, Italy; 2 Department of Biomedical and Biotechnological Sciences, University of Catania, 95123 Catania, Italy; 3 Department of Ophthalmology, University of Catania, 95123 Catania, Italy

**Keywords:** uveal melanoma, immune checkpoints, immunotherapy, CD47

## Abstract

Uveal melanoma (UM) represents the most frequent primary intraocular tumor, however, limited therapeutic options are still available. We have previously shown that cluster of differentiation 47 (CD47) is significantly upregulated in UM cells following inflammatory stimuli and that it represents a predictor of disease progression. Here, we aimed to better characterize the pathophysiological role of CD47 in UM. We show that CD47 is not modulated at different cancer stages, although patients with the lowest expression of CD47 show significant better progression-free survival, after correcting for the presence of BAP1, GNAQ, and GNA11 mutations. By stratifying patients based on the expression of CD47 in the tumor, we observed that patients with high levels of CD47 have a significant increase in immune score as compared to patients with low levels of CD47. In particular, deconvolution analysis of infiltrating immune cell populations revealed that a significantly higher number of CD4+ and CD8+ T cells can be found in patients with high CD47 levels, with the most enriched populations being the Th2, Treg, and CD8+ Tcm cells. We also show that a large number of transcripts are significantly modulated between the groups of patients with high and low levels of CD47, with a significant enrichment of interferon IFN-alpha regulated genes. The results from this study may propel the development of anti-CD47 therapies for UM patients.

## 1. Introduction

Uveal melanoma (UM) is the most common primary intraocular cancer in adults. It originates from the choroid in approximately 85% of the cases, while the remaining cases arise from the ciliary body (up to 8%) and the iris (up to 5%) [1]. A variety of both host and environmental factors influence the development of uveal melanoma. Significant risk factors have been described, including fair skin and light eye color, oculodermal melanocytosis, and cutaneous, choroidal, and iris nevus [2].

The most frequent chromosomal aberrations found in UM patients are chromosome 3 monosomy (that occurs in 50% of the cases) and amplification of 8q and 6p [1]. Monosomy 3 and polysomy 8q correlate with metastatic progression and fatal prognosis [3]. Indeed, chromosome 3 contains the tumor suppressor gene, BAP1; while in the 8q region, we can find tumor-promoting genes, including MYC (8q24) [4] and ASAP1 (DDEF1; 8q24) [5]. The liver accounts for 80–91% of the metastases. Although both cutaneous melanomas and uveal melanoma originate from melanocytes, their clinical behavior and underlying molecular mechanisms differ significantly [6]. For example, unlike cutaneous melanoma where metastasis to the central nervous system (CNS) occurs in 40–60% of cases, only 4–15% of uveal melanoma metastasizes to CNS. The reasons for this discrepant metastatic pattern have so far not been dismantled.

Immune-based therapies have improved the overall survival of cutaneous melanoma patients, but have failed to provide significant clinical benefits in unresectable/metastatic UM patients [7,8]. It is likely that since the eye is an immune privileged organ, the tumor and its metastases show local immune-evasive properties that affect the efficacy of immunotherapies [9,10]. Nevertheless, the increasing body of data on the immunobiology of UM may help to design of novel therapeutic strategies.

Cluster of differentiation 47 (CD47) is a cell membrane protein that inhibits macrophage phagocytosis, by binding the signal regulatory protein α (SIRPα) on Antigen-Presenting Cells (APCs). Downregulation of CD47 is observed on senescent or damaged cells and regulates their clearance by macrophage. In different tumor types, CD47 has been found to be upregulated and represents an independent negative prognostic factor [11,12]. We have previously shown that CD47 expression increases when UM cells are challenged with the supernatant from activated T cells and that higher levels of CD47 are associated to significantly lower disease-free survival time [9].

In the present study, we wanted to further characterize the pathophysiological role of CD47 in UM. The results from this study may push forward the design of anti-CD47 strategies for UM patients.

## 2. Results

### 2.1. Expression of CD47 and Correlation Analysis

**Figure 1:**
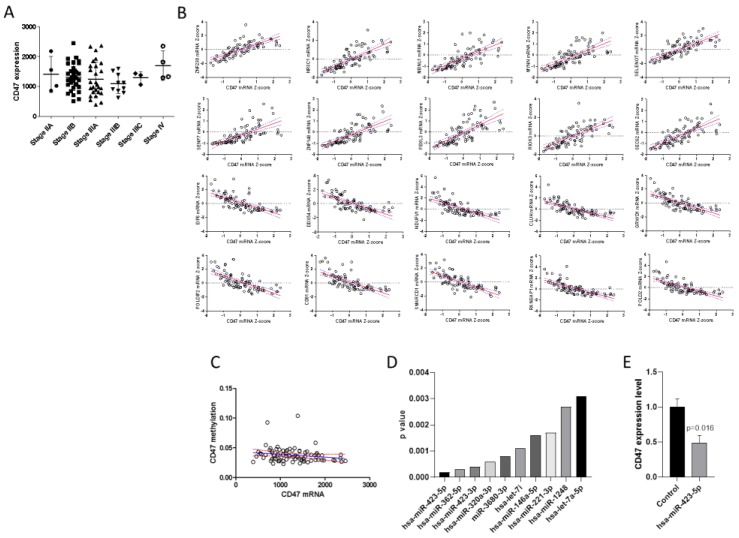
(**A**) Expression of cluster of differentiation 47 (CD47) in uveal melanoma (UM) samples at different TNM stages; (**B**) Top 10 positively correlated and negatively correlated genes to CD47 expression in UM samples; (**C**) Correlation between CD47 expression and methylation levels in UM samples (**D**) Top 10 miRNAs inversely correlated to CD47 expression levels as determined in the TCGA (The Cancer Genome Atlas) dataset; (**E**) Modulation of CD47 expression upon transfection of 92.1 cells with hsa-miR-423-5p mimic.

In order to determine the expression levels of CD47 among UM samples at different cancer stages, and to determine the potential mechanisms involved in the regulation of its expression, we interrogated the TCGA (The Cancer Genome Atlas) dataset. Overall, the dataset included 80 samples, respectively at stage IIA (n = 4); stage IIB (n = 32); stage IIIA (n = 27); stage IIIB (n = 10); stage IIIC (n = 3); and at stage IV (n = 4). As shown in Figure 1A, the transcriptomic levels of CD47 were not significantly different between cancer stages.

Analysis of the genes statistically correlated to CD47 identified 554 genes positively correlated (r > 0.7 and q value < 0.0001) and 54 negatively correlated (r < −0.7 and q value < 0.0001) (Figure 1B shows the correlation analysis for the top 10 positively and top 10 negatively correlated genes).

Correlation analysis between CD47 mRNA levels and methylation beta-values showed no correlation between CD47 expression and DNA methylation levels (Figure 1C).

Figure 1D shows the top 10 miRNAs inversely correlated to CD47 in the UM samples from the TGCA dataset. The most significant miRNA resulted to be hsa-miR-423-5p (Figure 1D). Accordingly, transfection of the 92.1 UM cell line with hsa-miR-423-5p mimic determined a significant downregulation of CD47 expression (Figure 1E).

### 2.2. Transcriptomic Differences between Samples with High and Low Levels of CD47

**Figure 2:**
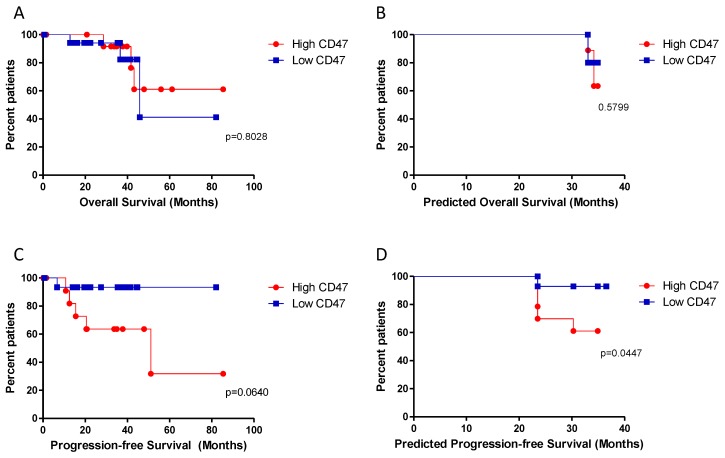
(**A**) Kaplan–Meier curve for overall survival in patients with different CD47 expression levels; (**B**) Kalan–Meyer curve for overall survival in patients with different CD47 expression levels after correcting for the presence of BAP1, GNAQ, and GNA11 mutations; (**C**) Kaplan–Meier curve for progression-free survival in patients with different CD47 expression levels; (**D**) Kaplan–Meier curve for progression-free survival in patients with different CD47 expression levels after correcting for the presence of BAP1, GNAQ, and GNA11 mutations.

Patients were stratified based on the transcriptomic levels of CD47, and survival curves were constructed for overall survival and progression-free survival. Although no differences were observed for overall survival (Figure 2A,B), patients with low levels of CD47 showed a trend of better progression-free survival (*p* = 0.064) (Figure 2C) that reached the statistical significance after correcting for the presence of BAP1, GNAQ, and GNA11 mutations (*p* = 0.0447) (Figure 2D).

**Figure 3:**
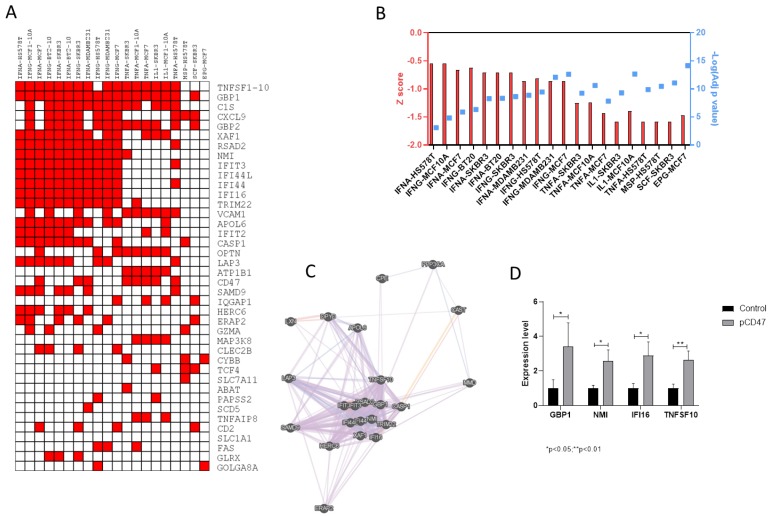
(**A**) Clustergram of Single Ligand Perturbation analysis for the upregulated genes in patients with high CD47 expression levels as compared to patients with low CD47 expression levels; (**B**) Histogram plot for Single Ligand Perturbation analysis for the downregulated genes in patients with high CD47 expression levels as compared to patients with low CD47 expression levels showing Z score and significance of the top 20 categories; (**C**) Gene network analysis of IFN-alpha-related genes differentially expressed in patients with high CD47 expression levels vs. patients with low CD47 expression levels; (**D**) PCR analysis of selected genes in 92.1 cells overexpression CD47.

Analysis of the transcriptomic differences between patients with high and low CD47 levels identified 334 significantly modulated transcripts, shared between the TGCA dataset and the validation set (Figure 3A). EnrichR analysis for the upregulated genes showed a significant enrichment for genes modulated by IFN-alpha (adj. *p-*value < 0.0001) and IFN-gamma (adj. *p-*value < 0.0001) (Figure 3B). No significant enrichment was instead observed for the downregulated genes (data not shown). Gene network analysis performed for the IFN-alpha-related genes identified GBP1, NMI, IFI16, and TNFSF10 as central hub genes (Figure 3C). Accordingly, overexpression of CD47 in 92.1 cells was associated to a significant increase in the levels of GBP1, NMI, IFI16, and TNFSF10 (Figure 3D).

### 2.3. Deconvolution Analysis

**Figure 4:**
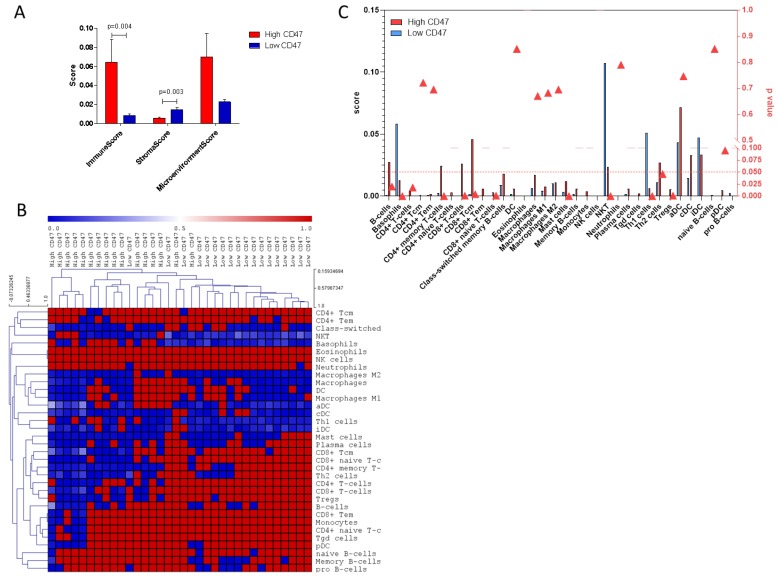
(**A**) Deconvolution analysis showing differences in immune score, stroma score, and microenvironment score between patients with high CD47 expression levels as compared to patients with low CD47 expression levels; (**B**) Heatmap showing the results of the deconvolution analysis for the infiltrating immune populations in patients with high CD47 expression levels as compared to patients with low CD47 expression levels; (**C**) Histogram plot showing the results of the deconvolution analysis for the infiltrating immune populations in patients with high CD47 expression levels as compared to patients with low CD47 expression levels. Red triangles indicate the *p*-value for differences between the two patients’ populations. The red dotted line indicates the threshold of significance for the *p*-value (*p* = 0.05).

Deconvolution analysis of cell populations in the UM samples revealed a significantly higher stroma score in samples with low CD47 levels as compared to samples with high CD47 expression (*p* = 0.003) (Figure 4A). On the contrary, a significantly higher immune score was found in samples with high CD47 levels as compared to samples to low CD47 levels (*p* = 0.004) (Figure 4). In particular, a significantly higher number of CD4+ and CD8+ T cells can be found in samples with high CD47 expression, with a significant increase in Th2, Treg, and CD8+ Tcm cells (Figure 4B,C). Similar data were obtained from the deconvolution analysis of the validation set of UM data, constructed using the GSE22138 and the GSE27831 datasets (Supplementary Figure S1). Indeed, the majority of the cell populations showed to be consensually modulated in the two analyses, with the only exception for CD4+ Tem, Memory B cells, iDCs (immature Dendritic Cells), naïve B cells, and CD8+ naïve T cells, which instead showed an opposite behavior in the two datasets. Moreover, none of these latter populations, but CD8+ naïve T cells, were significantly altered between patients with high and low CD47 levels in either of the two datasets (Supplementary Figure S1).

## 3. Discussion

Cluster of differentiation 47 (CD47) is a ~50 kDa glycosylated protein of the immunoglobulin superfamily, comprising an Immunoglobulin variable region (IgV)-like domain at the N-terminus, five membrane-spanning segments, and an alternatively spliced cytoplasmic C-terminus domain [13]. CD47 interacts with several ligands, including thrombospondin-1 and signal regulatory protein alpha (SIRPα), thus modulating several biological processes, such as cell migration, T cell activation, and cytokine production. Recent studies have highlighted a role for CD47-SIRPα in inhibiting phagocytosis. SIRPα is a member of the immunoglobulin superfamily that is expressed at high levels in the myeloid-lineage cells, i.e., macrophages and dendritic cells. The engagement of SIRPα by CD47 leads to the phosphorylation of SIRPα cytoplasmic immunoreceptor tyrosine-based inhibition (ITIM) motifs, the consequent recruitment of the Src homology phosphatases SHP-1 and SHP-2, the prevention of myosin-IIA accumulation and therefore, the inhibition of phagocytosis.

Increasing body of evidence shows the overexpression of CD47 in a variety of tumors, e.g., anaplastic thyroid carcinoma, esophageal squamous cell carcinoma, glioblastoma, T cell lymphoblastic leukemia, and bladder and breast cancer. Furthermore, the expression of CD47 on cancer stem cells (CSCs) suggests a role in cancer recurrence (reviewed by [13]).

We have previously proposed a possible role for CD47 in the immune evasive features of UM [9]. In particular, we have shown that CD47 is significantly upregulated by UM cells following inflammatory stimuli and that it represents a good independent predictor of disease progression.

In the present study, we aimed to better characterize the pathophysiological role of CD47 in UM. The whole-genome expression data has been largely used [14] to identify pathogenic pathways and therapeutic targets in several diseases, including autoimmunity [15,16,17,18,19,20], cancer [7,9,21,22], liver fibrosis [23], and neurodegenerative and infectious diseases [24] targets.

We first show here that there are no significant differences in CD47 levels between samples at different cancer stages, although patients with the lowest expression of CD47 show significant better progression-free survival, after correcting for the presence of BAP1, GNAQ, and GNA11 mutations. However, the reason why CD47 expression does not affect overall survival, while affecting progression-free survival, still needs to be deciphered. Nevertheless, a systematic review by Prasad et al. [25] showed only a weak correlation between anticancer drug-related changes in progression-free survival and overall survival, and a recent meta-analysis on FDA-approved oncologic immunotherapy demonstrated that progression-free survival benefits often do not correspond to overall survival benefits [26]. It is noteworthy that progression-free survival is a composite measurement as it includes both mortality and time-to-progression data, therefore, patient-level data should be ideally used to account for outcome correlations and dependency.

By stratifying patients based on the expression of CD47 in the tumor, we observed that samples with high CD47 levels have a significant increase in immune score as compared to samples with low CD47 expression. In particular, deconvolution analysis of infiltrating immune cell populations revealed that a significantly higher infiltrate of CD4+ and CD8+ T cells can be associated to high CD47 levels, with the most enriched populations being the Th2, Treg, and CD8+ Tcm cells. We also show that a large number of transcripts are significantly modulated between groups of samples diverging for CD47 expression, with a significant enrichment of IFN-alpha regulated genes. Our data are in line with those from Robertson et al. [27], who have identified 4 UM subsets, in which one cluster corresponded to Monosomy 3 (M3)-UM with immune infiltration. They have also shown that infiltrating CD8 T cells were present in 30% of M3-UM samples, while absent in the Disomy 3 (D3)-UM group, and that genes associated to the interferon-gamma signaling (IFNG, IFNGR1, and IRF1), lymphocyte migration (CXCL9 and CXCL13), cell-mediated cytolysis (PRF1 and GZMA), and immune-regulation (IDO1, TIGIT, IL6, IL10, and FOXP3) pathways were coexpressed with CD8a, highlighting the involvement of the immune microenvironment in the aggressive phenotype of some UMs, for instance the M3-UM subset.

In the last years, immune-based therapies have proved to be able to improve the overall survival of cutaneous melanoma patients, however, have failed to provide significant clinical benefits in unresectable/metastatic UM patients [28]. For instance, in a trial using either pembrolizumab, nivolumab, or atezolizumab, only 3.6% UM patients showed partial responses and 8.9% presented a stable disease [29]. Similarly, in a retrospective study on 82 UM patients who received ipilimumab, only 5% had an objective response and 29% had stable disease exceeding 3 months.

Since both cutaneous melanoma and UM arise from the same precursor, other factors are likely responsible for the different responses to immunomodulatory therapies. Therefore, effort has to be put in order to shed light on the immune evading mechanisms of UM, which may in the future help to design novel therapeutic strategies.

The data previously generated, along with the information from the present study, strongly support the development of anti-CD47-based therapy for UM patients, as it may represent a promising strategy to treat cancer and to increase survival. Several CD47 inhibitors are currently available, e.g., Hu5F9-G4, CC-90002, TTI-621, NI-1701, NI-1801, and SRF231, and some of them are already being tested in clinical trials on solid and hematological tumors. Recently, a Phase 1b study involving patients with relapsed or refractory non-Hodgkin’s lymphoma showed that the anti-CD47 antibody, Hu5F9-G4, combined with rituximab, exerted promising activity in patients with aggressive and indolent lymphoma, without showing clinically significant safety events [30]. These data support the potential use of combination therapies targeting CD47 and other inhibitory checkpoints, such as PDL-1 (Programmed death-ligand 1) and CTLA4 (cytotoxic T-lymphocyte-associated protein 4).

Moreover, as the receptor of CD47, SIRPα can also be targeted to inhibit the CD47–SIRPα pathway and recently, the monoclonal antibody KWAR23, which binds human SIRPα, given in combination with tumor-opsonizing monoclonal antibodies, greatly increased the cell-dependent killing of both hematopoietic and nonhematopoietic human cancer cell lines [31]. Finally, decoy receptors targeting CD47 have been developed, e.g., TT1-621, that has been shown to augment macrophage-mediated phagocytosis of hematologic and solid cancer cells [32].

The evidence provided by studies from our and other groups highlight the complex transcriptomic pattern that fine-tunes the interaction between UM cells and the immune cells and, therefore, additional efforts directed toward the investigation of focused immunotherapeutic approaches in patients with UM not responding to conventional treatments are strongly required.

## 4. Materials and Methods

### 4.1. Dataset Selection and Analysis

RNA Seq data and methylation data were obtained from the TCGA datasets through the cBioportal web-based utility (http://www.cbioportal.org; http://bit.ly/2s2Z0Qb). The dataset comprised 80 primary tumors, with no neoadjuvant therapy prior to excision. Clinical data included TNM disease stage, progression-free survival time, overall survival time, presence of BAP1, GNAQ, and GNA11 mutations. For each patient, the corresponding miRNA expression profile was obtained from the UCSC Xena Browser (https://xenabrowser.net/).

Patients were stratified in quartiles based on the expression of the CD47 gene. Differentially expressed genes between patients in the upper and lower quartiles were identified using the following parameters: q-value < 0.05 and log | fold change | >1. Ligand perturbation data were obtained via the EnrichR web-based utility [33]. EnrichR is a comprehensive server that integrates gene function, ontology, pathways, and statistical tools that enable to analyze genome-wide data from sequencing, proteomics, and gene expression experiments.

Gene network analysis was performed using the GeneMANIA web-based software [34]. Coexpression, Physical and Genetic interactions, Pathways, Colocalization, Shared Protein Domain, and Predicted Interactions were considered for the construction of the network. In the network, genes are represented by nodes and relationships as edges. Nodes with the highest number of interacting genes represent hubs in the network.

### 4.2. Cell Culture and Transfection

The UM cell line 92.1 was kindly provided by Prof. D. Tibullo (University of Catania). Cells were cultured in RPMI 1640 medium (Invitrogen, Carlsbad, CA, USA) supplemented with 10% fetal bovine serum (FBS; Gibco, Grand Island, NY, USA) in a humidified cell incubator with an atmosphere of 5% CO_2_ at 37 °C.

The miR-423-5p mimic (Life Technologies, Monza, Italy) was transfected into cells, using Lipofectamine RNAiMAX kit (Invitrogen) at about 50% cell confluency, following manufacturer’s instruction. The media was changed 24 h post-transfection and at 72 h post-transfection, total RNA was extracted and CD47 expression levels evaluated by real-time PCR.

In order to generate a CD47 overexpression construct, the ORF (Open Reading Frame) sequence of CD47 was cloned into the pcDNA3.4 vector (Life Technologies, USA). Transient transfection of 92.1 cells with the DNA plasmid encoding for CD47 or the empty plasmid was performed using Lipofectamine 2000 and Opti-MEM (Life Technologies), following manufacturer’s instructions. At 72 h post-transfection, total RNA was extracted and the expression levels of selected genes were evaluated by real-time PCR. Two micrograms of total RNA was reverse-transcribed using the High-Capacity cDNA Reverse Transcription Kit (Applied Biosystems, Monza, Italy) in a 20 μL reaction volume, and real-time PCR was performed using the SYBR Green PCR Master Mix (Applied Biosystems, Monza, Italy), 200 nM forward, 200 nM reverse primers, and 20 μg cDNA. Primers were in-house designed or obtained from PrimerBank (https://pga.mgh.harvard.edu/primerbank/) [35]. Gene expression was calculated using the formula: 2^−ΔΔCt^, where ΔΔCt = (C_t target gene_ − C_t beta-actin_) stimulated cells − (C_t target gene_ − C_t beta-actin_) control cells.

### 4.3. Computational Deconvolution of Infiltrating Immune Cells

In order to evaluate the proportions of the infiltrating immune cell subsets in UM samples diverging for the expression of CD47, we performed a computational deconvolution analysis. To this aim, we have used the web-based utility, xCell (http://xcell.ucsf.edu/) [36], a computational tool that is able, by using gene signatures, to infer the presence in a sample of various cell types, including active DC, astrocytes, B cells, CD4+ naive T cells, conventional DC, DC, memory B cells, plasma cells, Th1 cells, and monocytes.

### 4.4. Validation Set

In order to validate the data generated using the TGCA dataset, the two whole-genome expression microarray datasets GSE22138 and the GSE27831 were used. Both datasets were retrieved from the Gene Expression Omnibus database (GEO; https://www.ncbi.nlm.nih.gov/gds). GSE22138 included the transcriptomic profile of 63 UM samples from enucleation of untreated patients, while GSE27831 consisted of gene expression profiles of 29 UM patients. Both datasets were generated using the Affymetrix Human Genome U133 Plus 2.0 Array. Data were preprocessed following the Robust Multichip Analysis (RMA) procedure. In order to integrate the gene expression profiles from the two datasets, data were further normalized by performing Z score transformation [37], which allows the comparison of microarray data independently of the original hybridization intensities, within the same array type. When multiple probes hybridized to the same gene, the probe showing the highest variance among samples was considered. Overall, the resulting dataset included 91 primary UM samples and expression levels of 23,513 unique genes.

### 4.5. Statistical Analysis

Gene expression differences among samples at different cancer stages were evaluated using one-way ANOVA. Correlation analysis was performed using the nonparametric Spearman’s test. Survival analysis was performed using Kaplan-Meier and its significance analyzed by log-rank (Mantel–Cox) test. Predicted values were obtained by Multivariate General Linear Model using BAP1, GNAQ, and GNA11 mutations as fixed factors. For the analysis, a *p*-value < 0.05 was considered statistically significant. Statistical analysis was performed with GraphPad Prism 5 (GraphPad Software, San Diego, CA, USA) and SPSS 24 (IBM SPSS Statistics, IBM Corporation, Armonk, NY, USA).

## Supplementary Materials

The following are available online.
