# Supplementary figures and images for: Characterization of the Pathophysiological Role of CD47 in Uveal Melanoma
Source: Molecules. 2019 Jul 4;24(13):2450. doi: 10.3390/molecules24132450 (PMC6651482; doi:10.3390/molecules24132450)

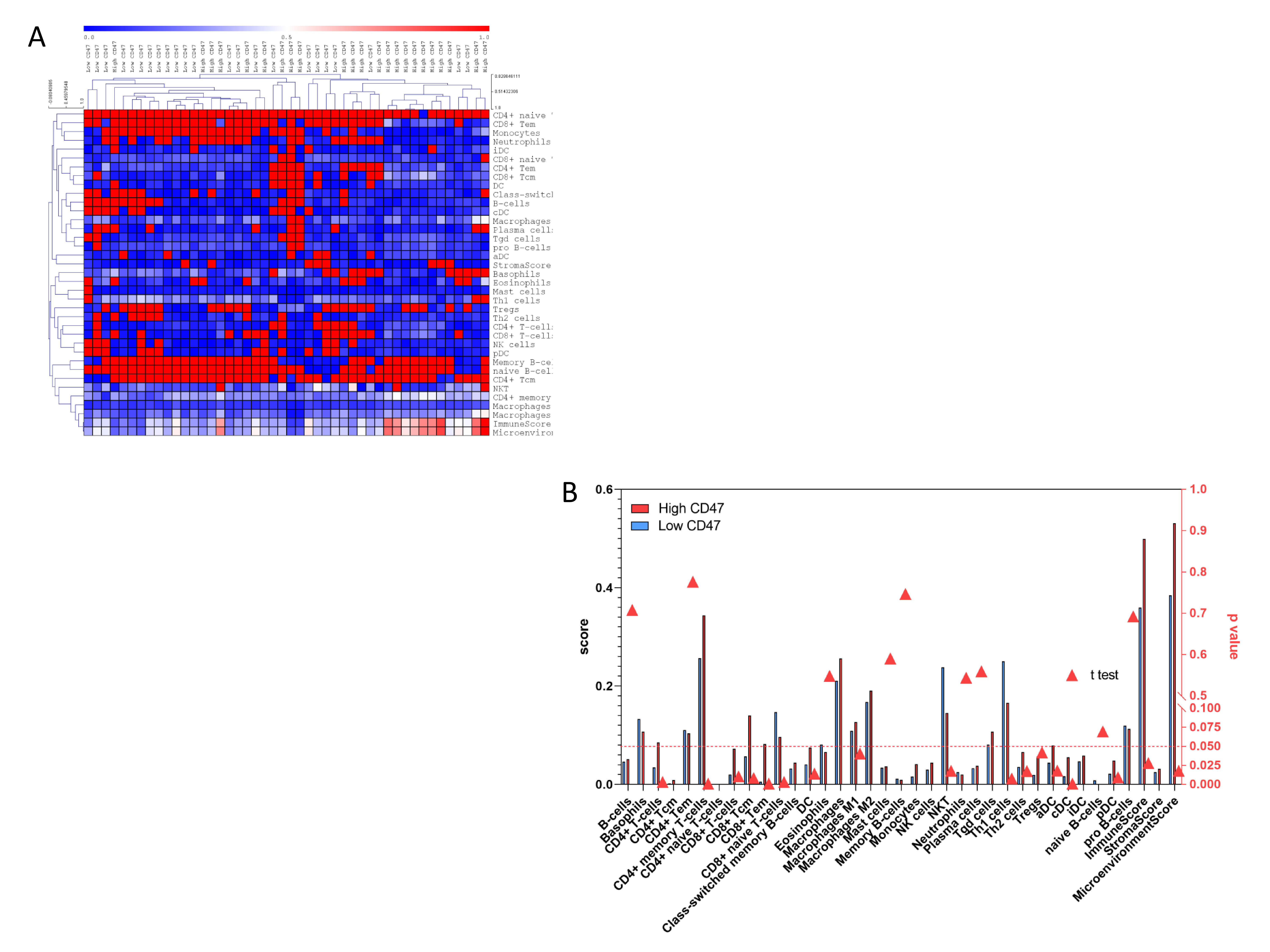

Supplement: Supplementary file 1 [file molecules-24-02450-s001.zip › molecules-512320-supplementary.tif]
